# The Role of Neuroinflammation in Glaucoma: An Update on Molecular Mechanisms and New Therapeutic Options

**DOI:** 10.3389/fneur.2020.612422

**Published:** 2021-02-04

**Authors:** Teresa Rolle, Antonio Ponzetto, Lorenza Malinverni

**Affiliations:** ^1^Eye Clinic, Department of Surgical Sciences, University of Torino, Torino, Italy; ^2^Department of Medical Sciences, University of Torino, Torino, Italy

**Keywords:** glaucoma, neuroinflammation, microglia, astrocytes, target, therapy, microbiome

## Abstract

Glaucoma is a multifactorial optic neuropathy characterized by the continuous loss of retinal ganglion cells, leading to progressive and irreversible visual impairment. In this minireview, we report the results of the most recent experimental studies concerning cells, molecular mechanisms, genes, and microbiome involved in neuroinflammation processes correlated to glaucoma neurodegeneration. The identification of cellular mechanisms and molecular pathways related to retinal ganglion cell death is the first step toward the discovery of new therapeutic strategies. Recent experimental studies identified the following possible targets: adenosine A_2A_ receptor, sterile alpha and TIR motif containing 1 (neurofilament light chain), toll-like receptors (TLRs) 2 and 4, phosphodiesterase type 4 (PDE4), and FasL-Fas signaling (in particular ONL1204, a small peptide antagonist of Fas receptors), and therapies directed against them. The continuous progress in knowledge provides interesting data, although the total lack of human studies remains an important limitation. Further research is required to better define the role of neuroinflammation in the neurodegeneration processes that occur in glaucomatous disease and to discover neuroprotective treatments amenable to clinical trials. The hereinafter reviewed studies are reported and evaluated according to their translational relevance.

## Introduction

Glaucoma is a chronic and progressive optic neuropathy characterized by death of retinal ganglion cells (RGCs) with consequent localized or diffuse thinning of the nerve fiber layer and increased cupping of the optic nerve head (ONH) (1). It is a social disease; its prevalence is believed to grow strongly, and about 80 million glaucomatous patients are expected in 2020 and 112 million in 2040 (2, 3). More than 7 million people worldwide are blind due to glaucoma, and the prevalence of bilateral blindness caused by this pathology varies from 6 to 16% in western countries (4).

The precise mechanisms leading to RGCs loss are not fully understood. Several studies have suggested that glaucoma has important analogies with other neurodegenerative pathologies correlated to inflammatory responses like amyotrophic lateral sclerosis, Alzheimer's disease, Parkinson's disease, Huntington's disease, and frontotemporal dementia (5–8).

The purpose of this minireview is to discuss the consolidated knowledge of neuroinflammation in glaucoma and to focus on new experimental evidences about possible therapeutic targets and latest treatment proposals.

## Main Actors Of Neuroinflammation in Glaucoma

### Microglia and Astroglia

Microglia and astroglia are the cell types involved in inflammatory responses within the retina (9); they consist of Müller cells and astrocytes and provide metabolic support of neurons, neurological regulation of ionic concentrations, and neuroprotective activities (8, 10–12). Microglial cells originate from blood monocytes migrating to the central nervous system (CNS) and present cellular antigens like CD11b/c and chemokine fractalkine receptor (CX3CR1) (13). They start from primitive erythromyeloid progenitors and mature in microglia (14) differentiating through different pathways dependent on colony-stimulating factor 1 receptor (CSF-1R) (15), interleukin-34 (IL-34) (16), and transforming growth factor-β (TGF-β) (17). After becoming mature, they take part in the inflammation process, which is activated by damage-associated molecular patterns (DAMPs) released by neural cells and also by astroglia and microglia (18–20). Among DAMPs, heat shock proteins (HSPs) are produced by RGCs when intraocular pressure (IOP) is elevated (21), while Tenascin-C is upregulated in astrocytes and induces toll-like receptor (TLR) activation (22). In response to the neuroinflammatory process, microglia release cytokines and chemokines (22–24) such as complement factors, tumor necrosis factor-alpha (TNF-α), and interleukin-6 (IL-6) that amplify the response and contribute to promote morphological changes of microglia into macrophages (25). M1 and M2 are two phenotypes of activated macrophages. M1 is proinflammatory and produces IL-1β, IL-12, and TNF-α (26); on the other hand, M2 synthetizes anti-inflammatory intermediaries such as IL-10, TGF-β, and neurotrophic factor insulin-like growth factor (IGF-1) (27–32).

Recently, in a study in DBA/2J mice with experimental glaucoma, the triggering receptor expressed on myeloid cells/TYROsine kinase binding protein (TREM/TYROBP) signaling network has been identified as the principal regulator mechanism of microglial responses to elevated IOP; infiltrating monocyte-like cells are likely responsible for the early proinflammatory signals (33).

Microglial cells communicate with astroglia via signaling proteins. Depending on this interaction, astroglia are differentiated into two types of reactive astrocytes, A1 and A2 (34–36). A1 has a detrimental effect (37); on the other hand, A2 has a neuroprotective function (38). Indeed, microglia and astroglia collaborate to regulate the inflammation process.

Different genes are implicated in the inflammatory pathways and are upregulated in the retina and ONH (39, 40). The first to be upregulated are TLR signaling pathways: for example, HSPs increase the expression of major histocompatibility complex (MHC) II and cytokine production (21). The second pathway is represented by nuclear factor-kappa B (NF-κB), which causes an increased expression of IL-1 cytokine family that promotes the cascade of inflammatory cytokines (TNF-α and IL-6). TNF-α is found in the optic nerve in glaucoma patients (41–43) and also Fas ligand (FasL), a proapoptotic protein, both being implicated in glaucoma pathogenesis (44). Recently, Oikawa et al. (45) demonstrated in feline's early glaucoma the upregulation of genes related to cell proliferation and immune responses (linked with the TLR and NF-κB signaling pathway), and they observed that proliferating cell types are different in ONH sub-regions. Microglia–macrophages were found in the prelaminar region and in the lamina cribrosa, while oligodendrocytes were more numerous in the retrolaminar region.

## Molecular Mechanisms

After reporting the fundamental concepts related to the neuroinflammation cells and the genes involved, we now refer to the latest studies on the main molecular mechanisms selected according to their presence in neurodegenerative diseases and their translational relevance.

The *exosomes* have a demonstrated role in neurodegenerative diseases. In an experimental glaucoma model (46), the exosomes, produced by naive microglia (BV-2 cells) in a condition of elevated hydrostatic pressure (BV-Exo-EHP, e.g., high IOP), induced the activation of retina microglia, production of cytokines, hypermotility, proliferation, and increase of phagocytosis. They promoted increase of reactive oxygen species and cell death, causing a reduction of retina ganglion cell number. The translation relevance of this study is that the exosomes own an autocrine function in the neuroinflammation pathway correlated to neurodegeneration.

Necroptosis, a recently discovered genetic form of cell death, has a fundamental role in neurodegenerative diseases (47). It is similar to necrosis with cell swelling, granular cytoplasm, chromatin fragmentation, and cellular lysis. Necroptosis differs from apoptosis because the cell content moves into the extracellular matrix in a passive way through the altered cell membrane. Necroptosis is induced by TNF-α and also by Fas and TNF-related apoptosis-inducing ligand (TRAIL), interferons (IFNs), TLR signaling, and viral infection via DAI (DNA sensor DNA-dependent activator of IFN regulatory factor). Ko et al. (47) in a neuroinflammation model of experimental glaucoma proved that the axon degeneration is sterile alpha and TIR motif1 (SARM1)-dependent and is induced by TNF-α with a consequent oligodendrocyte loss and RGC death. The necroptosis perpetrator is mixed lineage kinase domain-like pseudokinase (MLKL) through the reduction of the axon survival factors nicotinamide mononucleotide adenylyltransferase 2 (NMNAT2) and stathmin 2 (STMN2) that inhibit SARM1 NADase action. TNF-α also activates SARM1-dependent axon degeneration in sensory nerve cells through a different necroptotic signaling mechanism.

Several recent studies have dealt with the topic of *SARM1 axon degeneration pathway*. The axon health is preserved by the balance between the pro-survival NMNAT2 and STMN2 and pro-degenerative molecules dual leucine zipper kinase (DLK) and SARM1 (48). Activated DLK reduces the concentration of protective factors (NMNAT2 and SCG10) and exposes axons to traumatic and metabolic damages; this event is also related to mitochondrial dysfunction that in an independent way reduces the NMNAT2 and SCG10 concentrations in the axons (49). The equilibrium between axon survival and self-destruction is strictly related to axonal NAD+ metabolism. Sasaki et al. (50) studied in cell cultures and *in vivo* the biomarker cADPR, which controls NAD+ levels via SARM1 and mobilizes calcium. SARM1 has a basal activity in normal conditions. After injury, the axon degeneration is preceded by SARM1-dependent rise in the amount of axonal cADPR, but the contribution of cADPR in degenerative mechanisms has not been proven. The mitochondrial dysfunction can produce an incomplete activation of SARM1 and could predispose the axons to neurodegeneration.

In the context of neuroinflammation, it is mandatory to report the interesting developments that have taken place in recent years regarding the relationship between *microbiome* and *glaucoma*. Chen et al. conducted a study in germ-free mice, demonstrating that the absence of gastrointestinal (GI) bacteria abolished the development of glaucoma (51). The immune mechanism involved in the pathogenesis of the neural damage is related to CD4+ T-lymphocytes that recognize HSPs. Bacterial HSPs cross-reacted with both mouse and human HSPs. High IOP values induced the passage of CD4+ T-cells in the retina, and these T-cells were to blame for the extended phase of neurodegeneration in glaucomatous disease. They recognized specifically both bacterial and human HSPs. The induction of neurodegeneration in glaucoma requires pre-exposure to microbial flora, either the GI and oral one (51). In the past century, the presence of elevated titers of antibodies directed against small HSPs and cross-reacting with human HSPs was demonstrated in glaucoma patients (52). It was reported that these antibodies exert cytotoxicity when directly applied to human retina (53). It was proposed that immunomodulation should become the basis of glaucoma therapy. However, the immunosuppressive therapy could be dangerous and sometimes burdened by serious side effects. One solution could be the identification of antigens triggering or enhancing the autoimmune mechanism(s) and their elimination, aiming at reducing the active aggression by autoimmune T-cells. Since the development of glaucoma was shown to be linked to the microbiome, some of its components are potentially curable, such as *Helicobacter pylori*, a Gram-negative flagellar bacterium present worldwide, in 20–90% of individuals. Only active *H. pylori* infection induces cellular immune responses against the nervous system, due to molecular mimicry and cross-reactivity with components of host nerves. A large meta-analysis was recently published by Doulberis et al. (54). When the diagnosis of *H. pylori* infection was performed by gastric biopsy, the odds ratio (OR) for an association was very high (5.4), with confidence interval of 3.17–9.2 (highly significant); the strong association diminished to an OR of 2.08 when only serum antibodies were measured. Similarly, also in glaucoma, the antigens recognized by the immune system were reported in quite a large number. Geyer and Levo (55) suggested that the therapy should be directed not only against the IOP but also against autoreactive lymphocytes, on the basis of the lack of neurodegeneration in experimental mice models after depletion of either B-cells and in particular T-cells directed against HSP-derived peptides (55). However, this might not be sufficient. Beutgen et al. in a recent review reported elevated levels of a variety of autoantibodies (autoAbs) both in systemic circulation and in aqueous humor of glaucoma patients (56): not only anti-HSPs, but also against myelin basic protein and glial fibrillary acid protein. Such autoantibodies are typically involved in diseases of the nervous system.

## New Possible Targets and Proposals on Pharmacological Therapies

Hereinafter, the possible new therapeutic targets to reduce or block the neuroinflammation, which are more likely to be used in future clinical practice, are outlined. They are listed in alphabetical order. In Table 1, we provided more detail on new targets, their neuroinflammatory and neurodegenerative effects, and, where provided, the therapeutic options.

**Table 1:** New targets, their neuroinflammatory effects, impact of neurodegeneration and therapeutic options.

**Targets**	**Authors**	**Neuroinflammatory effects**	**Progression of neurodegeneration**	**Therapeutic options**
Adenosine A_2A_ receptor (A_2A_R)	Madeira et al. (57); Aires et al. (58)	Microglia activation: increase in mRNA levels of MHC-II, up-regulation of mRNA expression of TSPO, CD11b, TREM2	Synaptotoxicity, excitotoxicity, impaired retrograde axonal transport, axon degenerative profiles, disorganized and abnormal myelin wrapping, loss of RGCs	Caffeine
Astroglial NF-κB	Yang et al. (59)	Cytokine signaling, toll-like receptor (TLR) signaling, inflammasome activation	Neurodegeneration at different neuronal compartments: dendrite degeneration and synapse dysfunction, death of oligodendrocytes, RGC soma injury	Transgenic deletion of astroglial I_K_Kβ by pro-inflammatory cytokines
Endothelin-1 (ET-1)	Nor Arfuzir et al. (60)	Increase of IL-1β, IL-6 and TNF-α, NF-κB (activated by phosphorylation of I_K_Kβ), c-Jun (activated by JNK), STAT3 activation	Ischemia: retinal and optic nerve damage, NMDA induced excitotoxicity, induction of iNOS	Magnesium acetyltaurate (MgAT)
FasL-Fas signaling	Krishnan et al. (61)	Microglia activation: Induction of cytokines and chemokines (GFAP, Caspase-8, TNFα, IL-1β, IL-6, IL-18, MIP-1α, MIP-1β, MIP-2, MCPI, and IP10) complement factors (C3, C1Q) Toll-like receptor pathway (TLR4), inflammasome pathway (NLRP3), induction of apoptosis	Axon degeneration, RGCs death	ONL1204
Monocyte-like cells	Williams et al. (62)	Platelet adhesion, extravasation of the monocytes and enter the ONH (CD45hi/CD11b+/CD11c+)	Axon degeneration	DS-SILY, Itgam (CD11b, immune cell receptor)
ONH astrocytes (ONHAs)	Means et al. (63)	Activated caspases, Tau cleavage, NFTs formation, GFAP upregulation	ONHA dysfunction and degeneration, RGCs apoptosis	Polyphenolic phytostilbene and antioxidant *trans*-resveratrol (3,5,4′-trihydroxy-*trans*-stilbene)
PDE type 4 (PDE4) signaling	Cueva Vargas et al. (64)	Microglia activation: production of proinflammatory cytokines (TNFα, IL-1β, IL-6 and MIF), upregulation of TNFα/TNFR1 signaling, GFAP upregulation, increase of Iba1/CD68-positive cells	Axonal degeneration, low levels of cAMP-PKA	Ibudilast (inhibitor of cAMP phosphodiesterase type 4)
Sterile alpha and TIR motif containing 1 (SARM1)	Marion et al. (65); Krauss et al. (66)	TIR domain dimerization, NADase activation, reduction of NMNAT2, activation of calpains	Axonal degeneration	Biomarker Neurofilament light chain (NfL), therapeutic options not available yet
TLR2 and TLR4	Yang et al. (67); Ehlers et al. (68); Ji et al. (69)	Microglia activation: expression of GFAP and Iba-1, neuroinflammatory pathways TLR4-related	Production of inflammatory cytokines, leucocytes degranulation, modulation of neuroinflammatory response (local and systemic)	Alpha 1-antitrypsin (AAT) human umbilical cord mesenchymal stem cells (hUC-MSC) transplantation

### Adenosine A_2A_ Receptor (A_2A_R)

Madeira et al. (57, 70) demonstrated in a Sprague Dawley rats model of ocular hypertension (OHT) that *caffeine* (antagonist of adenosine receptors) administration prevents OHT-induced microglial activation and causes the modulation of retinal neuroinflammation and prevention of the RGCs loss. A recent study outlined the protective effect of microglial adenosine A_2A_ receptor (A_2A_R) blockade that can prevent, in human retina, microglial cell response to elevated IOP (58).

### Astroglial Nuclear Factor-Kappa B

The astroglial NF-κB was evaluated as a possible treatment target for the modulation of immune response in an experimental transgenic glaucoma mouse with deletion of IkappaB kinase beta (IκKβ) in astroglial cells (59). The study showed a reduction of the increase in proinflammatory cytokines (in particular TNF-α) with consequent protection of axon from degeneration and RGCs from apoptosis, as demonstrated also by pattern electroretinogram (PERG) data.

### Endothelin-1

Since 2012 (71), it was clarified that the increment of endothelin-1 (ET-1) in glaucomatous patients is linked to sub-clinical inflammation. Nor Arfuzir et al. (60), using Sprague Dawley rats that underwent intravitreal injection of ET-1, proved that the pre-treatment with magnesium acetyltaurate (MgAT) was protective against the increase induced by ET-1 in the retinal expression of IL-1β, IL-6, and TNF-α and against the activation of NF-κB and c-JUN induced by ET-1. MgAT promoted a higher RGC survival.

### FasL-Fas Signaling

FasL, a type II transmembrane protein of the TNF group, promotes apoptosis after binding to the Fas receptor and is involved in the pathogenesis of glaucoma also through inflammatory pathways. Krishnan et al. (61) demonstrated in microbead-injected wild-type (WT) mice that the treatment with *ONL1204*, a small peptide antagonist of Fas receptors, significantly reduced RGC death and loss of axons. The authors proved that ONL1204 blocks microglial activation and inhibits the induction of multiple genes involved in glaucomatous disease, as cytokines and chemokines (GFAP, caspase-8, TNFα, IL-1β, IL-6, IL-18, MIP-1α, MIP-1β, MIP-2, MCPI, and IP10), elements of the complement (C3 and C1Q), inflammasome pathway (NLRP3), and TLR4.

### Monocyte-Like Cells

Recently, in a mouse model of ocular hypertension (DBA/2J eyes), it was proved that monocyte-like cells enter the ONH and that monocyte–platelet interactions occur in glaucomatous tissue (62). Using these monocyte-like cells as therapeutic target, the authors demonstrated that the treatment with *DS-SILY*, a peptidoglycan that hinders the platelet adhesion to the vessel endothelium and to monocytes, and the treatment with genetic targeting of Itgam (CD11b), an immune cell receptor that blocks effusion of the monocytes from the vessels, can both be neuroprotective by reducing neuroinflammation.

### Optic Nerve Head Astrocytes

Means et al. (63) demonstrated that pretreatment with polyphenolic phytostilbene and antioxidant *trans-resveratrol* (3,5,4′-trihydroxy-trans-stilbene) on ONHs underwent oxidative stress with *tert*-butyl hydroperoxide (tBHP) and induced a significant reduction in activated caspases, neurofibrillary tangle (NFT) formation, and cleaved Tau. These findings outlined that resveratrol can have protective properties to prevent ONH astrocyte (ONHA) dysfunction and degeneration.

### Phosphodiesterase Type 4 Signaling

Cueva Vargas et al. (64) evaluated the anti-neuroinflammatory activity of ibudilast, a clinically approved cAMP phosphodiesterase (PDE) inhibitor with special affinity for PDE type 4 (PDE4). In an OHT rat model, the intraocular administration of ibudilast decreased the production of proinflammatory cytokines and reduced macroglia and microglial reactivity in the retina and optic nerve. Ibudilast had a positive effect on RGC soma survival, avoided axonal degeneration, and enhanced anterograde axonal transport in POAG eyes through activation of the cAMP/PKA pathway.

### Sterile Alpha and TIR Motif Containing 1

Since SARM1 induces Wallerian degeneration, this pathway could be a therapeutic target (65). The SARM1 inhibition prevents axonal degeneration in traumatic injuries and neurodegenerative disorders. As reported before, SARM1 is involved in innate immune response, and it is possible that the relationship between immune regulation and neurodegenerative disorders will play an important role for new therapeutic possibilities. The discovery of a reliable biomarker of axonal damage, i.e., the neurofilament light chain (NfL) and the possibility of testing NfL in plasma or serum, could be an important step to develop SARM1 inhibitors that protect axons from degeneration (66).

### Toll-Like Receptors 2 and 4

As reported, TLR signaling is involved in homeostasis and in pathology of CNS. TLR2 and TLR4 expressed by microglia take part in the glial response and in the neuroinflammation. Yang et al. (67) demonstrated that in C57BL/6J mice, the alpha 1-antitrypsin (AAT), a serine protease inhibitor, blocks microglial activation in chronic ocular hypertension model. It was shown that AAT inhibits leukocyte migration and has antithrombotic, antiapoptotic, and anti-inflammatory properties (68). In OHT rats, Ji et al. (69) demonstrated that the human umbilical cord mesenchymal stem cell (hUC-MSC) transplantation blocks TLR4-related microglial activation and neuroinflammatory pathways.

## New Proposal Holistic Approaches

In this section, we reported studies proposing holistic medicinal treatments to modulate/reduce the neuroinflammation in an experimental model of glaucoma. Table 2 illustrates new therapies with details on modulations of inflammatory mechanisms.

**Table 2:** New proposals of holistic treatments to contrast neuroinflammation in glaucoma.

**New therapies**	**Authors**	**Upregulation**	**Downregulation**
Antioxidants: Tempol	Yang et al. (72)	IL-13, IL-4, IL-6	Modulation of NF-κB signaling: decrease of TNF-α, IFN-γ, IL-1β, IL-2, IL-1α
Ketogenic diet	Harun-Or-Rashid et al. (73); Lu et al. (74); Shimazu et al. (75)	- Activation of Nrf2 - Increase of anti-inflammatory agents IL-4 and Arginase-1 - Increase of hydroxycarboxylic acid receptor 1 (HCAR1) - Increased levels of Arrestin β-2 protein, required for HCAR1 signaling - Increased GABAergic output and lowered presynaptic excitatory neurotransmitter release - Stimulation of HCAR2	Inhibition of - AMPK phosphorylation - Iba1 expression - NLRP3 inflammasome HCAR1-mediated - Oxidative stress by increased FOXO3A and MT2 activity - Class I histone deacetylases - NF-κB p65 nuclear translocation
Coriolus Versicolor and Hericium Erinaceus (Mushrooms)	Trovato Salinaro et al. (76)	IL-6, interferons immunoglobulin G, macrophages, T-lymphocytes, expression of Hsp70, TRX, HO-1	- Increase of Lipoxin A4 (endogenous eicosanoid) which blocks the production of pro-inflammatory mediators (ROS/RNS) - Stimulation NGF synthesis: modulation of cholineacetyltransferase + acetylcholinesterase
Hydrophilic Saffron extract (Crocus sativus)	Fernandez-Albarral et al. (77)	Inversion of OHT-induced down regulation of P2RY12	- Reduction of morphological signs of microglia activation - Reduction of various neurotoxic molecules (TNF-α, IL-β) - Suppression caspase-3 and caspase-9 activities: prevention of retinal ganglion cell death - Reduction of ROS: increase of blood flow in the retina and choroid

### Antioxidants

Oxidative stress can alter the immune function of the glia and promotes the neuroinflammation in glaucoma. The effects of the antioxidant Tempol were tested on ocular hypertensive retina and optic nerve samples and on NF-κB, a redox-sensitive transcriptional regulator of neuroinflammation. The analysis of markers of oxidative stress (proinflammatory cytokines, including IL-1, IL-2, IFN-γ, and TNF-α) demonstrated that the treatment was able to decrease the neuroinflammation markers (72).

### Ketogenic Diet

A recent research study (73) evidenced that the ketogenic diet reduces inflammation through inhibition of AMPK activation and HCAR1-mediated inhibition of the NLRP3 inflammasome. The way by which ketogenic diet works in neuroprotection is not completely known up to now. Researches supposed that ketogenic diet reduces oxidative stress, inhibits class I histone deacetylases, promotes Nrf2 activation to upregulate antioxidants, and inhibits NF-κB to reduce inflammation (74, 75).

### *Coriolus* and *Hericium* Nutritional Mushrooms

Trovato Salinaro et al. (76) reported the potential effect of *Coriolus* and *Hericium*, nutritional mushrooms, in the therapy of neurodegenerative diseases including Alzheimer's disease and glaucoma; they act as modulators of mechanisms of cellular protection (antioxidant properties) from mitochondrial dysfunction and neuroinflammation.

### Hydrophilic Saffron Extract

Studies conducted (77) in a mouse model of laser-induced unilateral OHT on a hydrophilic saffron extract (crocin 3%) demonstrated the reduction of morphological features of microglial activation in OHT and in contralateral eyes. The treatment with saffron extract in part inverted the downregulation of P2RY12. The saffron extract administrated orally protected RGCs from death by reducing neuroinflammation correlated with increased IOP.

## Discussion

**Figure 1:**
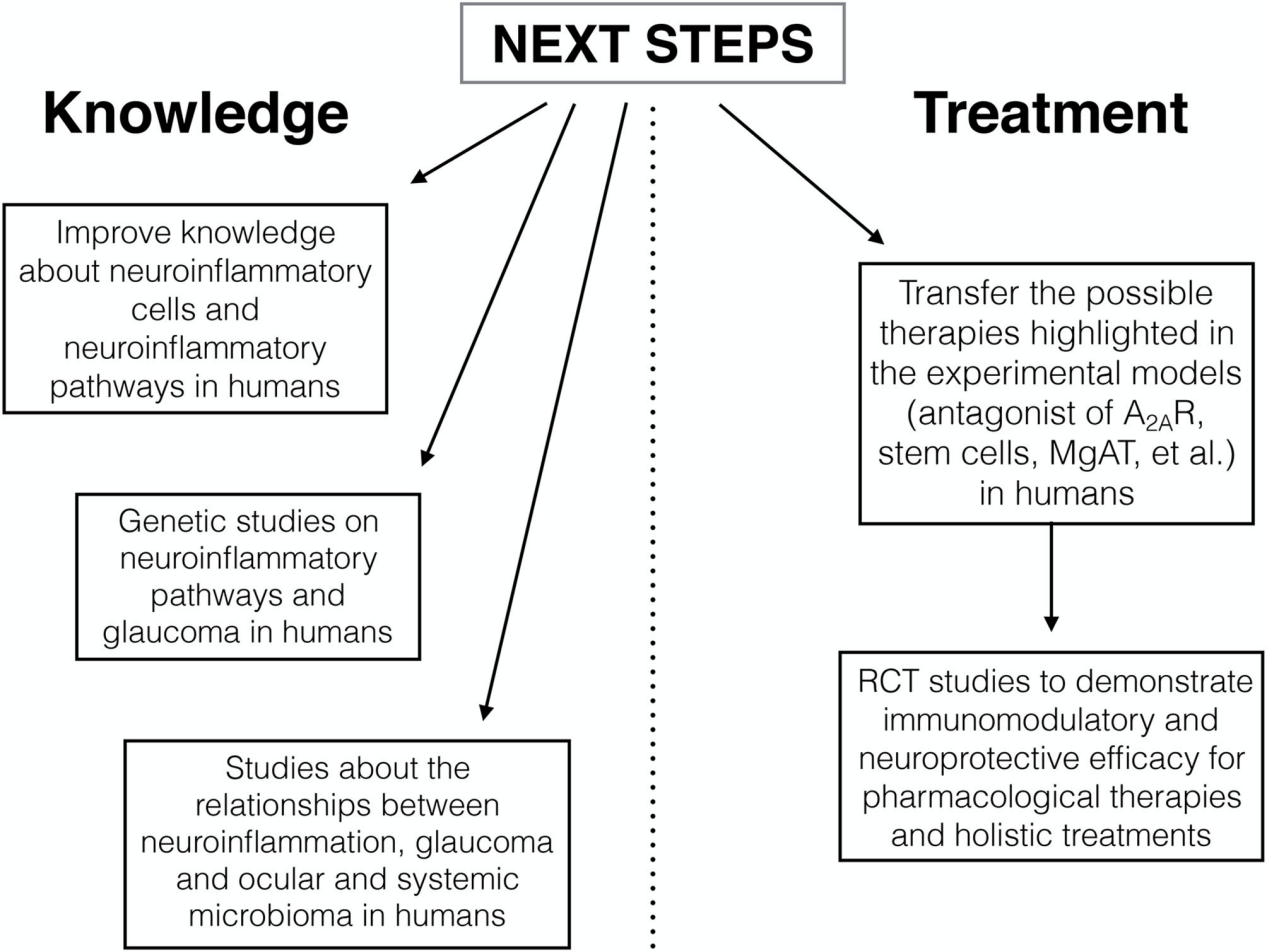
Next steps of developing future research.

The co-participation of the innate immune response and the inflammation in the pathogenesis of optic nerve degeneration in glaucomatous disease is now an established and proven event (78–82). The purpose of this minireview was to outline potential targets for future therapies, some of which are already being studied, and to describe possible treatments related to alternative medicine. These recent studies performed on animal models have improved our knowledge on cellular mechanisms, partly already known, but mainly on the signaling pathways of neuroinflammation. The studies analyzed openly the way to new possibilities of modulation of neuroinflammation (such as antagonist of A_2A_R, stem cells, MgAT, resveratrol, and alpha 1-antitrypsin) with a view to achieve effective neuroprotective treatments for glaucomatous disease. An important limitation is the almost total lack of researches in human. On the basis of the data reported, the possible ways of developing future research are illustrated in Figure 1. Further studies are needed to transfer the current experimental evidence into clinical research in order to slow down or to prevent the progressive glaucoma neurodegeneration.

## Author Contributions

All authors listed have made a substantial, direct and intellectual contribution to the work, and approved it for publication.

## Conflict of Interest

The authors declare that the research was conducted in the absence of any commercial or financial relationships that could be construed as a potential conflict of interest.
